# The Hospitals of Our Grandfathers, at Home and Abroad

**Published:** 1886-10-16

**Authors:** 


### THE HOSPITALS OF OUR GRANDFATHERS.

The Aggregate of Human Suffering.

HAPPILY for us all, none can fully realise the great aggregate of human suffering. But there are moments, perhaps, to most people when some faint and feeble conception of it flashes upon us, bringing with it a spasm of anguish and a sense of despair, which we are glad to shake off, as though it were some hideous nightmare. Such a moment I remember experiencing when, by the kindness of the Dean of St. Paul's, I was permitted to stand on the great dome, one summer night some years ago, to watch the pompous procession of the dawn out of the darkness, on to the rising of the sun. I remember well, as the cold grey of daylight broke upon the scene, looking across at what I took to be the roof of St. Bartholomew's Hospital, and at the fading glow of the lights in its windows, and for a moment there took possession of me a shuddering sense of all the horror of the place. I thought of the six or seven hundred beds beneath that roof there, and to my quickened imagination, to quote the words of Milton:

> "Dire was the tossing, deep the groans! Despair
> Tended the sick, busiest from couch to couch;
> And over these triumphant Death his dart
> Shook, but delay'd to strike, though oft invok'd
> With vows, as their chief good, and final hope."

I thought of the mangled limbs, the wasting diseases, the shattered constitutions—of the prayers that had been going up during the hours of darkness for the dawn that would bring no relief—of all the groaning, anguish, and writhing misery of all the hospitals in London. It was a painful inrush of thought, and for a moment the tragedy of human life weighed heavily upon me. I did not look far enough. From my giddy perch up there I ought to have peered out into the world beyond the sphere of hospitals and infirmaries, dispensaries and convalescent homes; and I ought to have looked back upon the slowly moving ages during which the Hospital, as we are familiar with it, was being gradually evolved. If I had done that, these institutions down there would have shone out as the very embodiment of all that was progressive and hopeful, enlightened, humane, and Christian, and even one's thoughts of the suffering within them would have been infinitely softened and relieved.

County Hospitals as they were.

If we dip a little into the history of the modern hospital, the majority of us will be amazed to find that not only are most of its characteristics of quite recent date, but that the very compassion which has created them, seems, not so very long ago, to have been quite in a rudimentary stage. Just imagine the kind of sensation which would be created nowadays, if some credible witness should pay an unexpected visit to a county hospital, and should find, amid surroundings of squalor and dilapidation, a poor emaciated creature tearing up the shirt from his back, to bandage up his own fractured thigh. That was the state of things existing less than a hundred years ago in one of our county hospitals. Floors were dirty, walls were black and filthy; in one part of the building the roof was open to the sky, and the beds in which the unfortunate patients were lying had nothing on them but filthy blankets, with not so much as a sheet to tear up for a bandage. No sheets in the house, no water. The diet comprised a threepenny loaf and two pints of milk, or rather milk and water. The surgery was a closet about ten feet by six feet. The furniture consisted of ten vials, some of them without corks, a little salve stuck on a board, some tow, and pieces of torn paper scattered on the floor. In another institution was found a state of things which induced a visitor to appeal to the treasurer. The poor suffering creatures in this precious hospital, he pointed out, had nothing but straw upon the floor, to lie upon, and what they had of that was dirty and insufficient, while they had scarcely so much as a blanket to cover them. "Let them find their own straw and blankets," said the treasurer.

The Hotel Dieu.

These institutions were, it is true, among the smaller and more remote of our hospitals, and one would hardly like to say very confidently what might not be found in some isolated instances even at the present time. But at the date we refer to, in many of the finest hospitals in Europe there were things to be met with which, to our more refined sensibilities, would be deemed horrible and revolting. For instance, the *Hôtel Dieu*, Paris, the oldest, and among the most famous institutions of the kind in the civilised world, presented features consistent neither with humanity nor the most rudimentary hygiene as we understand the subject. A humane law forbade the hospital to turn away any patient seeking admission; but the law, apparently, did not insist upon the provision of the necessary space. The whole establishment covered only about three acres, and within this area there were crowded at one time over five thousand sick and injured people. It was computed that, without allowing anything for staircases, passages, and offices of various kinds, there must have been a sick person in every floor-space of thirty feet right throughout the hospital—every floor-space seven feet and a half long and four feet wide, that is to say. A large proportion of the beds were intended to hold four people, and into these six patients were frequently crowded.

Three Small-Pox Patients in one Bed.

It is said to have been no unusual thing for two or three small-pox patients to have been huddled into a single bed, and as many as four women in childbirth were thus laid side by side. Indeed, if some accounts may be relied upon, things were at times far worse than this. The hospital seems to have been adapted only for times of normal public health. Any outbreak of epidemic disease crowded the place to a frightful extent. (*To be continued.*)

